# Circumventing the Crabtree effect: forcing oxidative phosphorylation (OXPHOS) via galactose medium increases sensitivity of HepG2 cells to the purine derivative kinetin riboside

**DOI:** 10.1007/s10495-020-01637-x

**Published:** 2020-09-21

**Authors:** Marta Orlicka-Płocka, Dorota Gurda-Wozna, Agnieszka Fedoruk-Wyszomirska, Eliza Wyszko

**Affiliations:** Institute of Bioorganic Chemistry, Polish Academy of Sciences, Noskowskiego 12/14, 61-704 Poznan, Poland

**Keywords:** Purine derivative, Kinetin riboside, Mitochondria, Crabtree effect, Metabolism, Cancer cells

## Abstract

Small-molecule compound-based therapies have provided new insights into cancer treatment against mitochondrial impairment. N6-furfuryladenosine (kinetin riboside, KR) is a purine derivative and an anticancer agent that selectively affects the molecular pathways crucial for cell growth and apoptosis by interfering with mitochondrial functions and thus might be a potential mitotoxicant. Metabolism of cancer cells is predominantly based on the Crabtree effect that relies on glucose-induced inhibition of cell respiration and thus on oxidative phosphorylation (OXPHOS), which supports the survival of cancer cells in metabolic stress conditions. The simplest way to circumvent this phenomenon is to replace glucose with galactose in the culture environment. Consequently, cells become more sensitive to mitochondrial perturbations caused by mitotoxicants. In the present study, we evaluated several cellular parameters and investigated the effect of KR on mitochondrial functions in HepG2 cells forced to rely mainly on OXPHOS. We showed that KR in the galactose environment is a more potent apoptosis-inducing agent. KR decreases the mitochondrial membrane potential, reduces glutathione level, depletes cellular ATP, and induces reactive oxygen species (ROS) production in the OXPHOS state, leading to the loss of cell viability. Taken together, these results demonstrate that KR directly acts on the mitochondria to limit their function and that the sensitivity of cells is dependent on their ability to cope with energetic stress.

## Introduction

Metabolism of most cancer cells relies on anaerobic glycolysis. Even in the presence of oxygen, mitochondrial respiration is suppressed, and this is a probable mechanism through which cells escape apoptosis [1]. Tumor cells are also able to shift the utilization of glucose and thereby promote increased lactate production instead of transporting pyruvate into the mitochondria, which was observed and reported for the first time by Otto Warburg [2, 3]. This inefficient fermentative mechanism for ATP generation provides precursors for many biosynthetic pathways and supports cancer cell proliferation, which is mainly the effect of “glycolytic phenotype” [4, 5] associated with the activation of oncogenic pathways [2]. However, like most cells within the body [6], hepatoma, breast cancer, and glioma cell lines were found to possess functionally efficient mitochondria where the majority of ATP is derived from oxidative phosphorylation (OXPHOS) [7, 8]. Recent studies on drugs inducing mitochondrial dysfunction have demonstrated that these cells are also able to switch their metabolic strategy alternatively, from fermentative to oxidative, and this phenomenon is termed as the Crabtree effect [8, 9]. This effect frequently accompanies the Warburg effect; however, in contrast to an anaerobic glycolysis, the Crabtree effect is usually referred to as a reversible, short-termed adaptive mechanism of cancer cells, which is based on glucose-induced inhibition of respiration, and therefore, OXPHOS is not only observed in proliferating tumor cells but may also occur in normal cells [10]. This phenomenon is also interpreted as a decrease in respiration together with the introduction of a glycolyzable substrate (e.g., glucose or other sugars). An increase in cellular ATP/ADP, a common intermediate for both glycolysis and OXPHOS, decreases the demand for respiratory flux to generate ATP [11].

Although the mechanism by which cancer cells undergo the Crabtree trigger remains unknown, it is assumed that many factors are involved in this switch. Metabolic plasticity is beneficial for cancer cells because it provides a microenvironment that promotes cell growth, especially in in vivo conditions. This might cause mitochondrial drug resistance and hinder with the testing of chemotherapeutics [12]. Mitochondria play a crucial role in the regulation of cellular energy metabolism, and they are also responsible for many other important functions such as induction of apoptosis, calcium redox, and homeostasis. Determination of the affected metabolic pathway might be strictly associated with the response of the mitochondria to treatment.

Under hypoxic and acidic conditions, glucose in culture medium forces growing cells to rely on glycolysis instead OXPHOS [13]. Even in the presence of efficient and functional mitochondria [6], the Crabtree effect occurs and supports cancer cells to survive in metabolic stress conditions. To avoid this phenomenon and to increase the significance of mitochondrial impairment in in vitro studies, it is very useful to adjust the cell culture environment, wherein cancer cells undergo enhanced OXPHOS rather than glycolysis, for example, by replacing glucose with galactose [13]. Many cancer and normal cell lines can easily adapt to new galactose culture conditions and start using OXPHOS for sufficient ATP generation [14]. The glucose withdrawal model has a wide range of applications, and it is particularly quite promising for studying bioenergetic pathways and mitochondrial-related processes. Although the glycolytic nature of tumor cells is a critical limitation, the galactose utilization strategy sensitizes cancer cells to putative mitochondrial toxicants [12]. Previous studies have shown that several mechanisms can lead to cancer cell death after glucose deprivation. The major pathways involved in the activation of apoptosis are ATP depletion [15, 16], induction of oxidative stress [17, 18], or both [15, 19].

The common mammalian cellular model used to identify mitochondrial dysfunction is HepG2. It is widely used as a primary model for investigating mitochondrial disturbances and for toxicity testing because of a large number of mitochondria and the ability to easily trigger metabolism according to the environmental conditions [12].

The present study aimed to demonstrate the influence of N^6^-furfuryladenosine (kinetin riboside, KR) on mitochondrial bioenergetics in HepG2 cells cultured in galactose medium. We attempted to confirm the complexity of the mechanism of action of KR and to determine whether mitochondrial cell death is the result of a cascade of unrelated events. Thus, we created an experimental pipeline to compare the effect of the compound in two different culture environments. The use of galactose allowed OXPHOS activation in HepG2 cells, which enabled to conduct experiments involving energy imbalance and induction of oxidative stress after KR treatment. KR has been recently reported to have a strong anticancer effect and may selectively influence various steps of intracellular signaling and molecular pathways that are crucial for cell growth, proliferation, and apoptosis [20]. KR is a representative of a large group of ribonucleoside analogs, which include the mimetic agent AICAR and 8-chloroadenosine that are well established and already used in clinical trials. Although both of them are purine derivatives, each may have a unique mechanism of action in cancer cells.

By using flow cytometry, confocal microscopy, and bioluminescent determination of ATP, we evaluated several cellular parameters such as inhibition of cell proliferation, apoptosis induction, cell energy phenotype and bioenergetic and oxidative imbalance. We investigated the effect of KR on mitochondrial functions in cells with forced mitochondrial OXPHOS. Here, we showed that KR is a potent apoptosis-inducing agent in a concentration-dependent manner. We also analyzed the apoptosis of two additional cell lines, namely T47D (breast cancer) and A172 (human glioblastoma), which underwent the Crabtree effect. Taken together, these results demonstrate that KR is a natural anticancer agent and might be a promising mitotoxicant that is comparable to drugs with known mitochondrial toxicity.

## Materials and methods

### Materials

Kinetin riboside, carbonyl cyanide 3-chlorophenylhydrazone (CCCP), and 2-deoxy-D-glucose (2-DG) were purchased from Sigma-Aldrich. 2-NBDG (2-(N-(7-nitrobenz-2-oxa-1,3-diazol-4-yl) amino)-2-deoxyglucose) was purchased from Thermo Fisher Scientific.

### Cell culture

Human hepatocellular carcinoma cells (HepG2) purchased from ECACC were grown in Eagle’s Minimum Essential Medium (EMEM) (ATCC) supplemented with 10% fetal bovine serum (FBS, Sigma-Aldrich) and antibiotics (ATCC). Human mammary gland cell line (T47D) purchased from ATCC was grown in RPMI-1640 medium (Sigma-Aldrich) supplemented with 10% FBS (Sigma-Aldrich) and antibiotics (Sigma-Aldrich). Human glioblastoma cell line (A172) was obtained from ATCC and grown in EMEM (ATCC) supplemented with 10% FBS (Sigma-Aldrich) and antibiotics (ATCC). All cell lines were cultured at 37 °C in 5% CO_2_ atmosphere. Both culture media, RPMI-1640 and EMEM, are referred to as low-glucose media because they contain 2 and 1 g/l of D-glucose, respectively. Cells grown in supplemented media without KR addition were used as negative control for all experiments.

### Galactose medium

Experimental medium, referred to as galactose medium, was Dulbecco’s Modified Eagle’s Medium (DMEM) supplemented with 10 mM galactose as the sole source of carbohydrate, 2 mM glutamine (Thermo Fisher Scientific), 5 mM HEPES (Gibco), 1 mM sodium pyruvate (Thermo Fisher Scientific), 10% FBS (Sigma-Aldrich), and antibiotics (Sigma-Aldrich). To change cellular metabolism into aerobic and increase OXPHOS, the growth medium was changed to galactose medium 16 h prior to the experiment. This time was sufficient to increase aerobic capacity. Subsequently, KR was added to the medium at an appropriate concentration, and the medium was incubated for 6 h.

### Real-time analysis of cell proliferation using the xCELLigence System

Impedance-based real-time cell proliferation analysis was performed using the xCELLigence system (Roche). Cell index (CI) and normalization values were recorded using the RTCA Software 1.2.1. In brief, 100 µl of supplemented glucose or galactose medium was added to E-plates for measuring background values, and HepG2 cells were then seeded in additional 50 µl medium at the density of 2 × 10^3^ cells per well. The cells were allowed to attach to the E-plates at 37 °C and 5% CO_2_ saturation in the cell incubator for 30 min prior to insertion into the xCELLigence platform. Cell density optimization was ensured by preliminary experiments. To induce metabolic switch, glucose medium was replaced with galactose for the next 16 h. After incubation, the cells were treated with KR at the final concentration of 20, 40, and 80 µM (based on the obtained results of IC_50_ value; data not shown) in additional 50 µl medium. Control cells were cultured in the supplemented medium alone. The real-time monitoring of the proliferation of HepG2 cells treated with KR was monitored at 30-min intervals from the time of plating for 120 h.

### Apoptosis/necrosis assay by flow cytometry

Apoptosis/necrosis assay was performed in HepG2 cells by double staining with CellEvent™ Caspase 3/7-FITC (Thermo Fisher Scientific) and 7-AAD (BD-Pharmingen) fluorescent dyes with excitation/emission at 503/530 nm and 546/647 nm, respectively. Briefly, the cells (4 × 10^5^) were seeded onto 6-well plates containing the growth medium (EMEM) and incubated until 70–80% confluency. Subsequently, the cells were grown in galactose medium for 16 h or in low-glucose medium supplemented with 20 mM 2-DG for 24 h. On the next day, the cells grown in galactose medium were treated for 6 h with KR at the final concentration of 20, 40, and 80 µM. The cells cultured with 2-DG were treated with KR for the next 24 h. The cells were then detached with trypsin (Thermo Fisher Scientific) and washed twice with 1 ml of DPBS (Thermo Fisher Scientific). Subsequently, the cells were harvested and suspended in a staining solution containing CellEvent™ Caspase 3/7-FITC (10 µM) and 7-AAD (1 µg/ml) for 30 min at 37 °C in dark. The cells were analyzed immediately after staining with excitation at 488 nm by Accuri C6 flow cytometer (Becton Dickinson).

### LIVE/DEAD analysis by confocal microscopy

HepG2 cells were seeded at the density of 3.5 × 10^4^ cells/well in 4-chamber glass bottom cell culture dishes (Grenier Bio-One) and cultured in EMEM at 37 °C and 5% CO_2_ saturation. Next, the medium was changed to galactose medium for another 16 h. Subsequently, the cells were treated for 6 h with 20, 40, and 80 µM KR and then analyzed by the LIVE/DEAD assay (Thermo Fisher Scientific). The assay discriminates live from dead cells by simultaneously staining in galactose medium with green-fluorescent 2 µM calcein-AM and red-fluorescent 4 µM ethidium homodimer-1 to indicate loss of plasma membrane integrity. Additionally cells nuclei were labeled with 5 µg/ml Hoechst 33342. Next, the cells were incubated for 15 min under growth conditions and subsequently washed three times with PBS to remove free dyes and then placed in FluoroBrite DMEM (Thermo Fisher Scientific).

Live cell imaging was performed using a Leica TCS SP5 confocal laser scanning microscope equipped with a White Light Laser (470–670 nm) and an environmental cell culture chamber that provided controlled conditions of temperature, CO_2_ saturation, and humidity. Sequentially scanned images were collected at 494/515 nm (± 20) and 517/602 nm (± 20) by using a Plan Apo 20/0.6× water/oil-immersion objective with 2× digital zoom. Leica LAS AF and LAS X software with 3D deconvolution module was used for imaging control and image processing, respectively.

### Mitochondrial membrane potential (ΔΨm) analysis by flow cytometry and confocal microscopy

HepG2 cells were seeded at the density of 4 × 10^5^ cells/well onto 6-well plates and cultured with EMEM at 37 °C and 5% CO_2_ saturation until 70–80% confluency was reached. Next, the medium was changed to galactose medium for another 16 h. Subsequently, the cells were treated for 6 h with 20, 40, and 80 µM KR. After incubation, the cells were detached with trypsin (Sigma-Aldrich) and washed twice with 1 ml of DPBS (Thermo Fisher Scientific), and alterations in ΔΨm were analyzed using the mitochondrial membrane potential (MMP)-sensitive cationic dye 5,5ʹ,6,6ʹ-tetrachloro-1,1ʹ,3,3ʹ-tetraethylbenzimidazolylcarbocyanine iodide (JC-1) (Thermo Fisher Scientific), which can selectively enter mitochondria and change color from red to green as the membrane potential decreases. The cells were stained with 2.5 µM JC-1 for 30 min at 37 °C in dark. Immediately after staining, the cells were analyzed with excitation at 488 nm by Accuri C6 flow cytometer (Becton Dickinson). The maximum emission wavelengths of JC-1 monomers and “J-aggregates” were ~ 525 (FL-1 channel) and ~ 590 (FL-2 channel) nm, respectively. Next, 50 µM CCCP was used to create a strong, single positive green fluorescence signal. Simultaneously, the MMP was analyzed using another potentiometric dye, namely TMRE (tetramethylrhodamine). For flow cytometric analysis, the cells were prepared as above and stained with 80 nM TMRE for 30 min at 37 °C in dark. The fluorescence intensity of TMRE was measured by flow cytometry in the lower quenching mode, with excitation and emission at 549 and 574 nm (FL2 channel), respectively. The mean fluorescence intensity representing the intracellular MMP level was analyzed with excitation at 488 nm by Accuri C6 flow cytometer (Becton Dickinson).

For imaging, HepG2 cells were seeded at the density of 3.5 × 10^4^ cells/well into 4-chamber glass bottom cell culture dishes (Grenier Bio-One) and cultured in EMEM at 37 °C and 5% CO_2_ saturation. Next, the medium was changed to galactose medium for another 16 h. Subsequently, the cells were treated for 6 h with 20, 40, and 80 µM KR, and the MMP change was then assessed using the lipophilic JC-1 dye. Next, the cells were rinsed with PBS, placed into a prewarmed (37 °C) fresh medium containing 3 µM of JC-1, and incubated for 15 min under growth conditions. Next, the cells were washed three times with PBS to remove free dyes and then placed in FluoroBrite DMEM (Thermo Fisher Scientific). Live cell imaging was performed using Leica TCS SP5 as described above. Sequentially scanned images were collected at 485/530 nm (± 20) and 535/590 nm (± 20) excitation/emission for JC-1 green fluorescent monomers and red fluorescent J-aggregates, respectively, by using a Plan Apo 20/0.6× water/oil-immersion objective with 6× digital zoom. Leica LAS AF and LAS X software with 3D deconvolution module was used for imaging control and image processing, respectively.

### Mitochondrial mass measurement by MitoTracker Green FM via flow cytometry

HepG2 cells were seeded at the density of 4 × 10^5^ cells/well onto 6-well plates, cultured in EMEM at 37 °C and 5% CO_2_ saturation, and incubated until 70–80% confluency was reached. Next, the medium was changed to galactose medium for another 16 h. Subsequently, the cells were treated for 6 h with 20, 40, and 80 µM KR. For determination of mitochondrial mass after KR treatment in glucose medium, the cells were incubated with 20, 40, and 80 µM KR for 24 h. Subsequently, glucose and galactose conditioned cells were detached with trypsin (Sigma-Aldrich), washed twice with 1 ml of DPBS (Thermo Fisher Scientific), and then incubated at 37 °C with 50 nM MitoTracker Green FM probe (Life Technologies) for 30 min in dark. MitoTracker Green FM (ex/em: 490/516 nm) is a green fluorescent mitochondrial stain that localizes to mitochondria regardless of the MMP. Immediately after staining, the cells were washed with DPBS and analyzed with excitation at 488 nm by Accuri C6 flow cytometer (Becton Dickinson).

### Oxidative stress measurements in HepG2 cells by flow cytometry

HepG2 cells were seeded at the density of 4 × 10^5^ cells/well onto 6-well plates, cultured in EMEM at 37 °C and 5% CO_2_ saturation, and incubated until 70–80% confluency was reached. Next, the medium was changed to galactose medium for another 16 h. Subsequently, the cells were treated for 6 h with 20, 40, and 80 µM KR. The intracellular reactive oxygen species (ROS) level was analyzed by staining with H_2_DCFDA (ex/em: ~ 492–495/517–527 nm) and PI (ex/em: 535/617 nm) according to the manufacturer’s protocol (Thermo Fisher Scientific), wherein fluorescence is triggered in the presence of ROS. The cells were detached by trypsin (Sigma-Aldrich) and washed twice with 1 ml of DPBS (Thermo Fisher Scientific). The supernatant was discarded, and the pelleted cells were suspended in 0.5 ml DPBS containing H2DCFDA and PI at the final concentration of 0.5 µM and 25 µg/ml, respectively. The cells were then incubated at 37 °C for 30 min in dark. The decrease in cellular GSH concentration was measured by staining with the nonfluorescent Thiolite Green dye (AAT Bioquest) that emits strong fluorescence upon reacting with thiols with excitation and emission at 540 nm and 590 nm. After KR treatment, the cells were detached with trypsin and washed twice as described above. Subsequently, the cells were suspended in a staining solution and incubated at 37 °C in 5% CO_2_ for 30 min. Immediately after staining, the fluorescence intensity was analyzed using Accuri C6 flow cytometer with excitation at 488 nm.

The above mentioned cell culture conditions were also used for measuring the mitochondrial oxidative stress (superoxide level) induction by flow cytometry. After incubation with KR, the cells were detached with trypsin and washed with DPBS (1 ml). Cell pellets were stained with MitoSOX (2.5 µM) for 10 min at 37 °C in dark.

For confocal microscopy analysis of mitochondrial superoxide, HepG2 cells were seeded at the density of 6 × 10^4^ cells/well into 4-chamber glass bottom cell culture dishes (Grenier Bio-One) and cultured at 37 °C and 5% CO_2_ saturation. Next, the medium was changed to galactose medium for another 16 h. Subsequently, the cells were treated for 6 h with 20, 40, and 80 µM KR. The cells were stained for 30 min with MitoSOX at the final concentration of 4 µM, rinsed with PBS, and placed into prewarmed (37 °C) FluoroBrite DMEM (Thermo Fisher Scientific). Fluorescence images of live cells were collected at 37 °C on a Leica TCS SP5 confocal laser scanning microscope with an environmental cell culture chamber. Sequentially scanned images were acquired using a Plan Apo 20× water/oil-immersion objective with excitation and emission spectra at 510 and 570–600 nm, respectively. Leica LAS AF and LAS X software with 3D deconvolution module was used for controlling scan process and image processing, respectively.

### Measurement of cellular ATP content

HepG2 cells were plated at the density of 4 × 10^5^ cells/well onto 6-well plates, cultured in growth medium (EMEM) at 37 °C and 5% CO_2_ saturation, and incubated until 70–80% confluency was reached. Subsequently, the cells were grown in galactose medium for 16 h and then treated for 2 h with 40 and 80 µM KR and simultaneously with 1 µM 5-iodotubercidin prior to KR treatment. After incubation, the cells were detached by trypsin, washed twice with 1 ml of DPBS, and centrifuged, and the pelleted cells were resuspended in 100 µl PBS. Cell lysates were prepared by freeze thaw cycles. Briefly, the cells were freezed at − 80 °C for 5 min and then thawed at 37 °C. This step was repeated three times. After centrifugation, cell debris was discarded, and the total protein concentration was measured spectrophotometrically at 280 nm. Quantitative determination of ATP (with recombinant firefly luciferase and its substrate D-luciferin) was performed using the Molecular Probes^®^ ATP Determination Kit (Thermo Fisher Scientific) according to the manufacturer’s protocol. Luminescence was measured at 560 nm using the Synergy2 BioTek plate reader.

### Monitoring of glucose uptake with 2-NBDG by flow cytometry

HepG2 cells (4 × 10^5^) were seeded onto 6-well plates and cultured in the supplemented medium (EMEM) until 70–80% confluency was reached. Subsequently, the cells were grown in galactose medium for 16 h and in low-glucose medium supplemented with 20 mM 2-DG for 24 h with KR. On the next day, the cells remaining in galactose medium were treated for 6 h with 20, 40, and 80 µM KR. The cells were then washed with PBS, added to culture media (low glucose when possible) supplemented with 100 µM 2-NBDG that shows excitation and emission maxima at 465/540 nm (a fluorescent glucose analog used to monitor glucose uptake in living cells as an indicator of cell viability; Life Technologies #N13195), and incubated for 1 h at 37 °C with 5% CO_2_. Subsequently, the cells were detached with trypsin (Thermo Fisher Scientific) and washed twice with 1 ml DPBS (Thermo Fisher Scientific). Stained cells were immediately analyzed with excitation at 488 nm by Accuri C6 flow cytometer (Becton Dickinson).

### Analysis of energy metabolism in HepG2 cells

HepG2 cells were seeded on XFp 8-well cell culture microplates (7.5 × 10^3^ cells/100 µl medium/well) overnight in glucose and galactose medium. The culture medium was removed from each well and replaced with 180 µl of Seahorse XF Base Medium containing 1 mM pyruvate and 2 mM glutamine, supplemented with 10 mM glucose or 10 mM galactose, and prewarmed to 37 °C. The cells were incubated in a CO_2_-free incubator at 37 °C for 1 h. Prior to rate measurements, the XFp Analyzer gently mixed the assay media in each well for 10 min to allow the oxygen partial pressure to reach equilibrium. The oxygen consumption rate (OCR) and extracellular acidification rate (ECAR) were measured simultaneously three times to establish a baseline rate.

The Agilent Seahorse XFp Cell Energy Phenotype Test Kit provides the compounds necessary to measure the metabolic phenotypes and metabolic potential of live cells by using 10 µM oligomycin (an inhibitor of ATP synthase) and 10 µM FCCP (a mitochondrial uncoupling agent). Two events occurred with a simultaneous injection of these stressor compounds.

### Statistical analysis

Each analysis consisted of three biological and two technical replicates. Statistical analysis was performed using GraphPad Prism version 6.00 for Windows (GraphPad Software, San Diego CA, USA). To determine the significance of flow cytometric analyses of MMP, oxidative stress, mitochondria number, and glucose uptake, one-way analysis of variance ANOVA followed by Tukey’s multiple comparison test was used. Significance of cell viability by apoptosis/necrosis staining was determined by two-way analysis of variance ANOVA followed by Bonferroni Correction. All results are expressed as a mean ± SD. P values < 0.05 were considered statistically significant. Measurement of ECAR and OCR was determined by two-tailed pairwise Student’s *t* test. Two groups were compared, and P values < 0.05 were considered to be significant.

## Results

### Replacing glucose with galactose in growth medium increases cell death after KR treatment

Our goal was to compare whether galactose medium, which forces and enhances OXPHOS, affects cell sensitivity to KR and provides greater and more rapid effect than glucose medium. Thus, we examined real-time cell proliferation by using the xCELLigence system and performed flow cytometric analysis of cell apoptosis, mitochondria localization assay, and glucose uptake monitoring with 2-NBDG. We started from the selection of an optimal concentration of KR that was sufficient to track induction of cell death in HepG2 culture (data not shown).

**Fig. 1:**
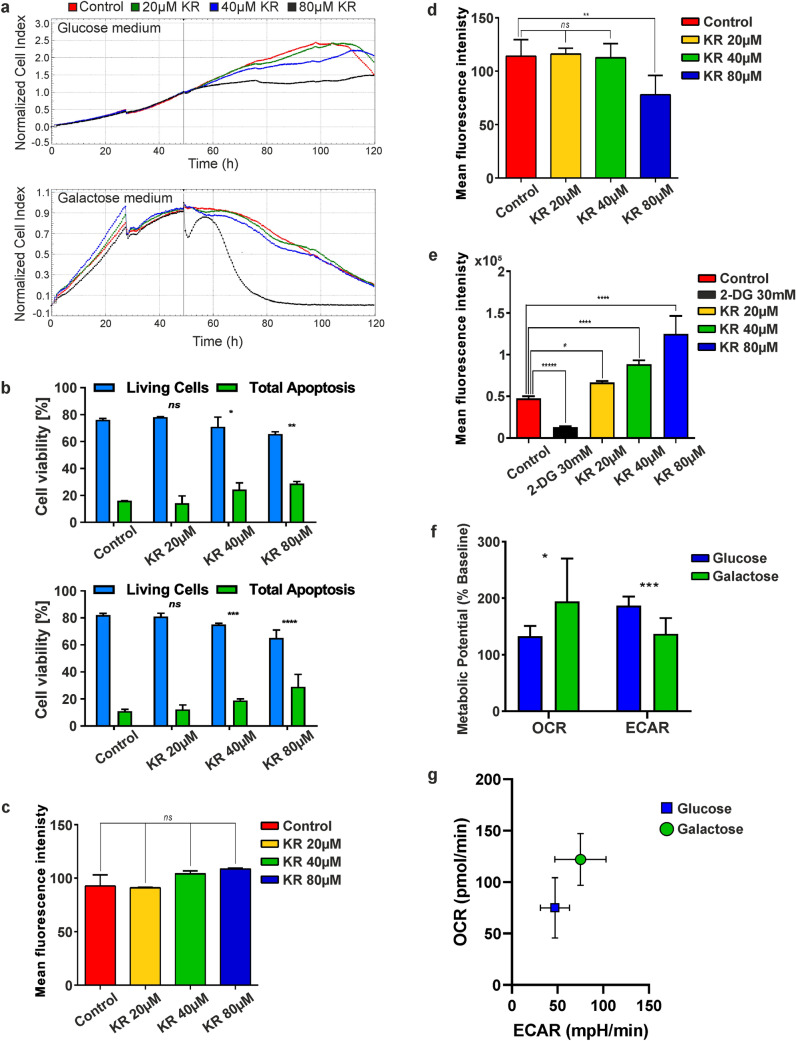
Kinetin riboside (KR) treatment of HepG2 cells is enhanced by replacement of glucose with galactose in the culture medium. **a** Real-time cell proliferation in glucose vs. galactose medium in the presence of KR. The influence of KR (20–80 µM) on HepG2 cell proliferation was monitored by the xCELLigence system for 120 h at 30-min intervals. The results are representative of at least three independent experiments. **b** Apoptosis/necrosis assay of HepG2 cells after KR treatment in glucose (upper graph) and galactose medium (bottom graph). The level of apoptosis was evaluated by flow cytometry using dual staining with Casp 3/7-FITC/7-AAD (Thermo Fisher Scientific). Glucose withdrawal sensitizes HepG2 cells to KR. Blue bars indicate live cells, whereas green bars represent both early and late apoptotic cells. Data are presented as a mean percentage of the total analyzed population (10,000 cells) ± SD from three independent experiments. **c, d** Measurements of mitochondrial mass using MitoTracker Green by flow cytometry in glucose and galactose medium respectively. Quantitative results of mitochondrial mass are representative of three independent experiments. **e** Measurement of uptake of a fluorescent deoxyglucose analog (2-NBDG) by flow cytometry in HepG2 cells in galactose medium. Cell viability was determined by the rate of glucose uptake by cells after KR treatment at different concentrations. To monitor glucose uptake by living cells, a fluorescent analog of glucose (2-NBDG) was used to determine mean fluorescence. Fluorescence intensity measured by Accuri C6 flow cytometer was plotted as bar graphs (mean ± SD) from three independent experiments. As a positive control, 2-deoxy-D-glucose was used. **f** Comparison of glycolytic and aerobic metabolism in the cells by evaluating OCR and ECAR parameters. **g** Metabolic map illustrating metabolic profiles of HepG2 cells cultured in glucose and galactose medium by OCR and ECAR values. Statistical significance is indicated with asterisks: (ns) p > 0.05, (*) p < 0.05, (**) p < 0.01, (***) p < 0.001, (****) p < 0.0001

The xCELLigence system allowed us to show that the Crabtree effect is a short-term response of cells to adverse environmental conditions. The cells cultured in galactose medium were more sensitive to the antimetabolite KR. Because of its toxicity in the experimental conditions, the analysis time was reduced from 24 to 6 h, except for real-time proliferation assay. We used the xCELLigence system for monitoring HepG2 cell proliferation to compare the toxicity of KR in culture media supplemented with glucose or galactose. Viability of HepG2 cells was monitored for 120 h at 30-min intervals (Fig. 1a). The kinetics of cell proliferation measurement provided the temporal information on the toxicity of the tested compound. Cell index values are influenced by several parameters such as cell number, cell size, cell–substrate interaction, and cell–cell attachment. Therefore, the xCELLigence system uses impedance measurements for real-time monitoring of cell growth and death.

We observed a significant decrease in the Cell Index value of HepG2 cells after 24 h treatment with KR in galactose medium, whereas inhibition of cell proliferation in glucose was less remarkable (Fig. 1a). This indicated that HepG2 cells were sensitive to both conditions, but the rate of response was different.

Apoptosis and necrosis are two major processes leading to cell death. To investigate whether KR-induced apoptosis in HepG2 cells, we performed flow cytometric analysis by dual staining with CellEvent^®^ Caspase-3/7 Green ReadyProbes^®^ Reagent and 7AAD. KR affected HepG2 proliferation in a dose- and time-dependent manner (Fig. 1b). After 6 h of incubation with 20–80 µM KR in galactose medium (bottom graph), the percentage of apoptotic cells (Caspase-3/7/7AAD-positive) increased to 35.5% compared to control cells (11.92%), whereas in glucose medium (24 h treatment with KR; upper graph), the effect was less pronounced and twice lower (Fig. 1b). To determine the number of mitochondria within HepG2 cells growing in galactose and glucose medium, we used MitoTracker Green FM, which localizes in mitochondria regardless of Δψ. MitoTracker Green FM allows to measure mitochondrial mass and has emerged as a useful tool to evaluate mitophagy. Low cellular toxicity of KR was observed in cells growing in glucose medium, which was evidenced by the lack of fluorescence intensity shift and no change in mitochondria amount (the number of mitochondria slightly increased to approximately 17% after KR treatment; Fig. 1c). In galactose medium, a slight decrease in fluorescence intensity was observed after exposure to 20–40 µM KR (Fig. 1d), while after 80 µM, the decrease in fluorescence intensity was significant and correlated with the decrease in mitochondria number by 31.5% compared to the control cells.

Cellular metabolism comprises many metabolic reactions that are involved in the conversion of the carbon source (nutrient uptake) into building blocks needed for biosynthesis. As the metabolite flux increased, forced by glycolysis, cancer cell proliferation was enhanced by providing more energy. Thus, we used the fluorescent analog of glucose, namely 2-NBDG, followed by flow cytometric detection for real-time quantification of glucose uptake by cells treated with KR growing in both media (Fig. 1e). The effect was observed only in HepG2 cells growing in galactose medium. The higher the KR concentration, the more pronounced was glucose uptake, and this finding correlated with the increase in fluorescence to more than 2.5-fold in 80 µM KR-treated cells compared to the control cells. The appearance of glucose in galactose medium immediately forced glycolysis and diminished OXPHOS metabolism after supplementation with KR. In glucose medium, no changes were observed (data not shown) in cells treated with an inhibitor of glycolysis, namely 2-DG.

We also characterized the metabolic phenotype of HepG2 cells using a Seahorse extracellular flux (XFp). In this system, mitochondrial respiration (OCR) is used to evaluate OXPHOS and extracellular acidification (ECAR) as a glycolysis indicator. We compared cells growing in glucose and galactose medium, and we found that in cells cultured in galactose medium, OXPHOS levels increased by approximately 46% and the activity of ECAR was 26.8% lower than that in glucose-cultured cells. This might be an evidence that HepG2 cells growing in galactose medium increase their oxygen consumption capacity via enhancing OXPHOS (Fig. 1f, g).

### Glucose withdrawal enhances the effect of KR on different cell lines exhibiting the Crabtree effect

**Fig. 2:**
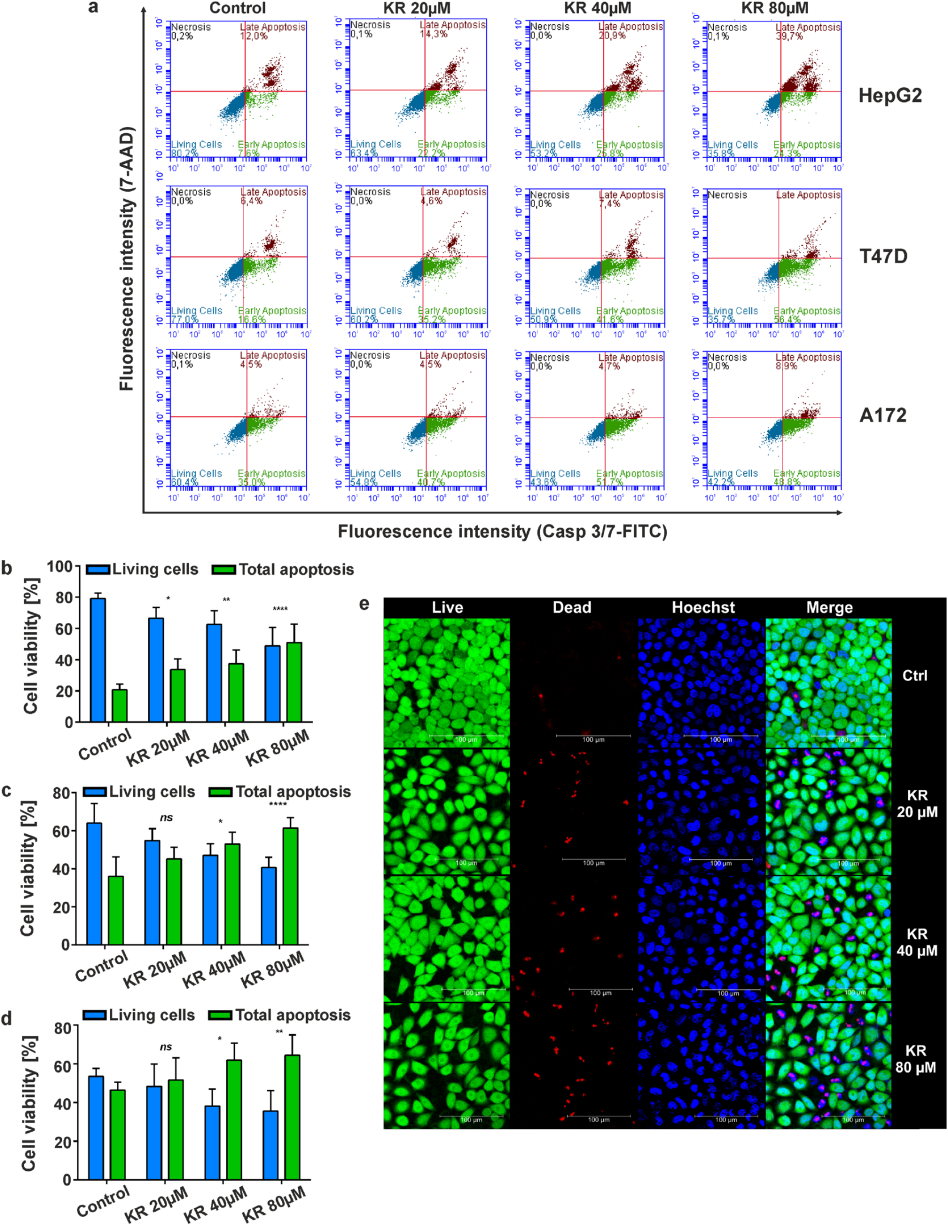
Comparison of the effect of KR in galactose medium on cell survival of three different cell lines showing the Crabtree effect. **a** Level of apoptosis was evaluated in HepG2, T47D, and A172 cell lines by dual staining with Casp 3/7-FITC/7-AAD. The percentage of live and apoptotic cells after 6 h of KR treatment at different concentrations is presented in a diagram. **b–d** Blue bars indicate live cells, whereas green bars represent both early and late apoptotic cells. Data are presented as a mean percentage of the total analyzed population (10,000 cells) ± SD from three independent experiments. Statistical significance is indicated with asterisks: (ns) p > 0.05, (*) p < 0.05, (**) p < 0.01, (****) p < 0.0001. **e** Confocal microscopy analysis of the viability of HepG2 cells in galactose medium 6 h after KR treatment using the LIVE/DEAD assay (Thermo Fisher Scientific)

We assessed whether KR can induce apoptosis in T47D (human mammary gland cell line) and A172 (human glioblastoma cell line) cells growing in galactose medium, as these cells were also undergoing the Crabtree effect (Fig. 2a). Flow cytometry analysis indicated that the apoptotic effect of KR on both cell lines is similar to that observed in HepG2 cells. The percentage of apoptotic HepG2 cells treated with 80 µM KR increased by 44.4% compared to the control cells, while it increased by 33.4% and 18.2% in T47D and A172 cells, respectively (Fig. 2a–d). These results confirmed that apoptosis is involved in the KR-induced inhibition of cell proliferation in galactose medium. Thus, we showed that KR is a potent apoptosis-inducing agent in a concentration-dependent manner. Comparison of the apoptotic effect of KR among different cell lines undergoing the Crabtree effect showed that the effect of the compound depends on cell type (Fig. 2a). Confocal microscopy analysis of HepG2 cell viability in galactose medium after 6 h KR treatment showed the distribution of living (green) and dead (red) cells (Fig. 2e). Increasing level of dead cells was correlated with the increasing concentrations of KR.

### Inhibition of glycolysis with 2-DG in HepG2 cells treated with KR is less efficient in inducing cell death than in cells exposed to KR cultured in galactose medium

**Fig. 3:**
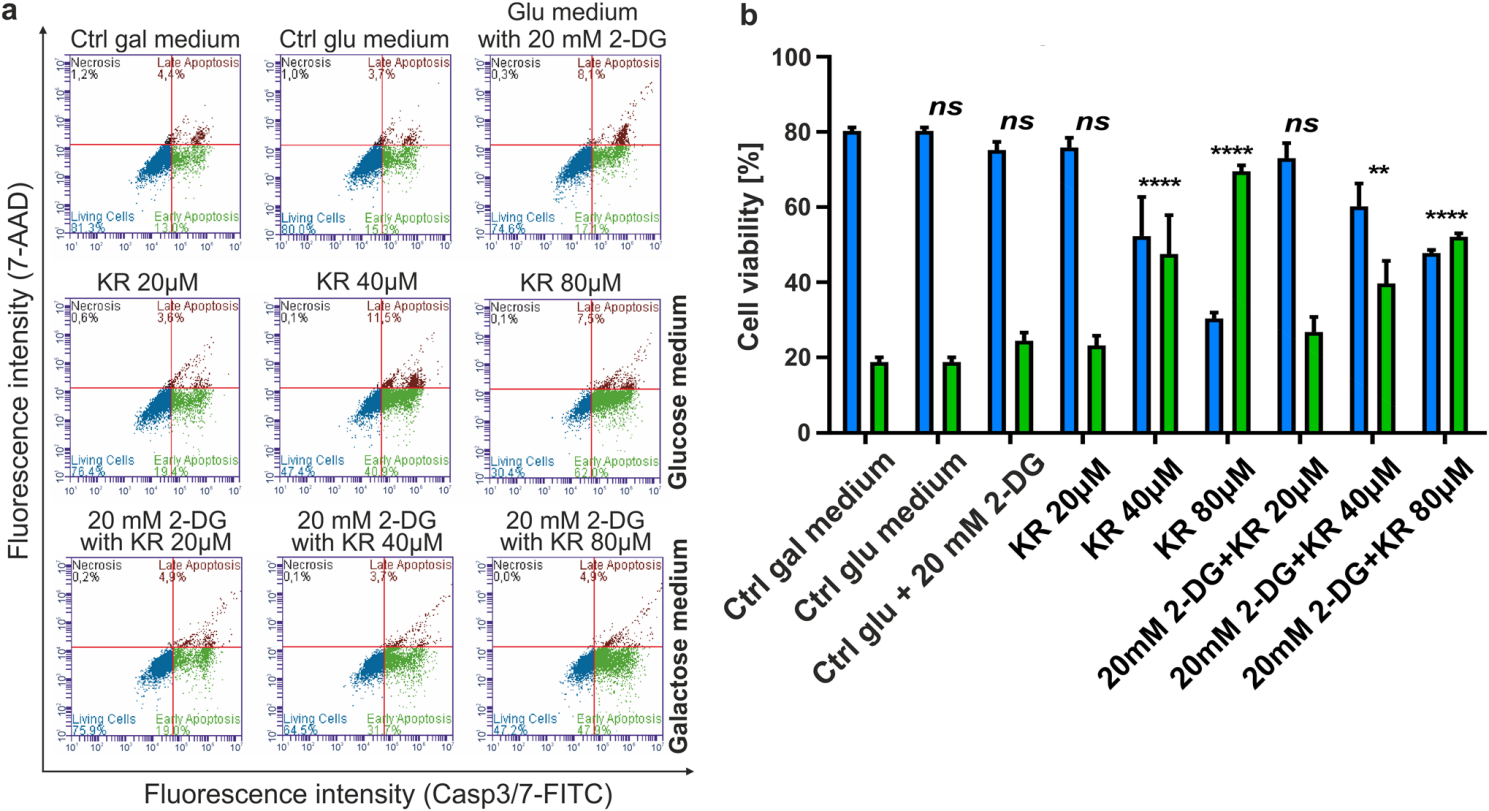
Influence of 2-deoxy-D-glucose on the viability of HepG2 cells after KR treatment. Comparison of cell viability after KR treatment (6 h) cultured in galactose medium and with the addition of 2-deoxy-D-glucose. **a** Level of apoptosis was evaluated by flow cytometry and presented as a representative diagram of live, early apoptotic, and late apoptotic cells. **b** The data are also presented on a bar graph, where blue bars indicate live cells and green bars indicate both early and late apoptotic cells. Data are shown as a mean percentage of the total analyzed population (10,000 cells) ± SD from three independent experiments. Statistical significance is indicated with asterisks: (ns) p > 0.05, (**) p < 0.01, (****) p < 0.0001

To determine and confirm which of the metabolic pathways is affected by KR, we performed apoptosis/necrosis assay in HepG2 cells growing in galactose or glucose medium supplemented with the glycolytic disruptor 2-DG (Fig. 3). 2-DG blocks the first step of glycolysis (it acts to competitively inhibit the production of glucose-6-phosphate at the hexokinase level). Inhibition of glycolysis decreases the concentration of pyruvate, which integrates the tricarboxylic acid cycle (TCA) to produce ATP. Pyruvate pool was however maintained by the exogenous pyruvate in the growth medium.

Cells in galactose medium were treated with KR for 6 h, whereas cells whose main energetic substrate is glucose were incubated for the first 24 h with the glycolysis inhibitor 2-DG and then with KR for the next 24 h. We observed that the apoptotic effect of KR is faster and more effective in cells growing in galactose medium with enhanced OXPHOS. The percentage of apoptotic HepG2 cells treated with 80 µM KR increased up to 52.1% compared to the control cells, while this percentage was up to 33.6% in cells growing in 2-DG conditions (Fig. 3a, b). The 2-DG inhibitor was not toxic to the cells, but when KR was added, apoptosis was observed, which was not as significant as that observed in HepG2 cells growing in galactose medium (Fig. 3).

### KR induces MMP perturbation and reduces ATP level in HepG2 cells growing in galactose medium

**Fig. 4:**
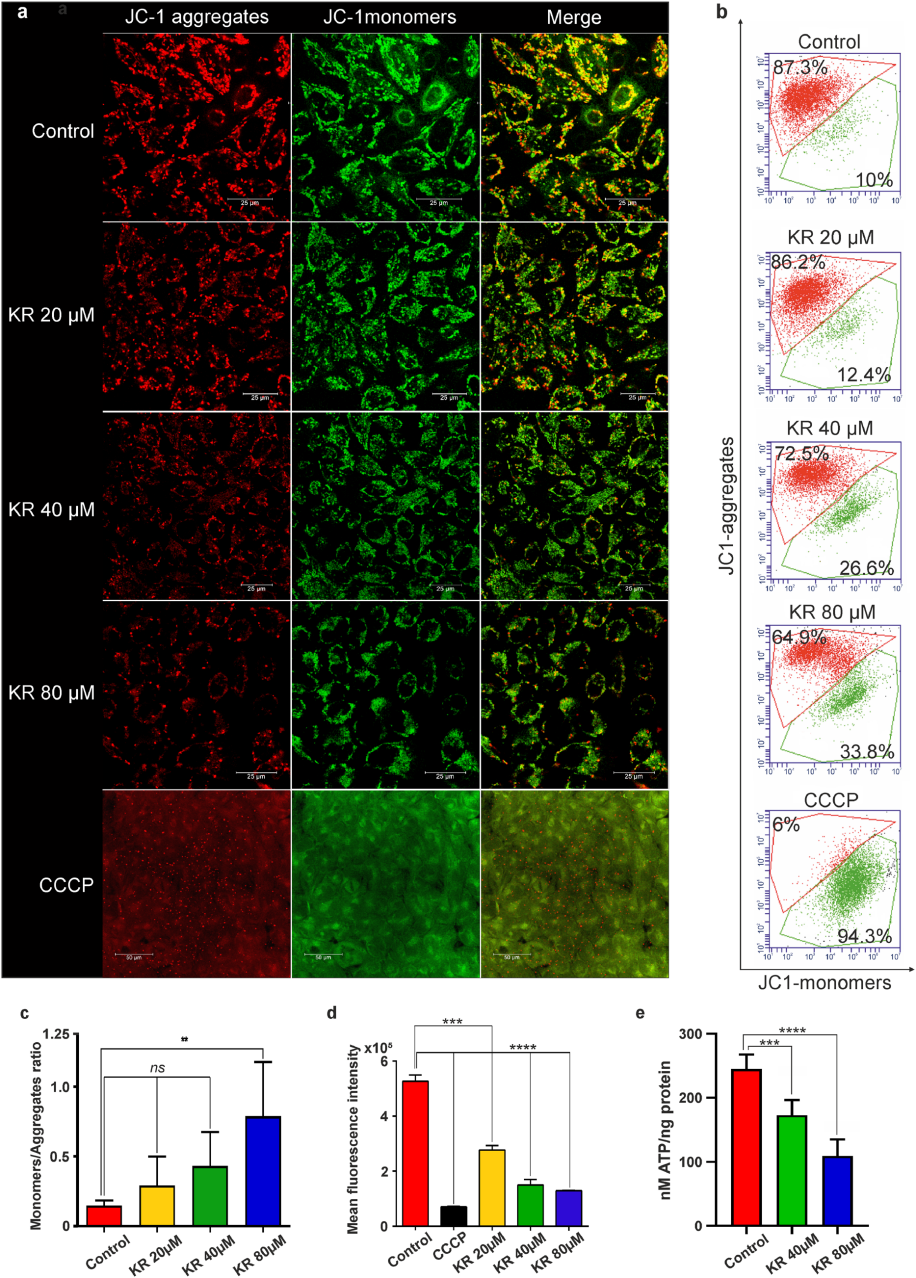
Bioenergetic status of HepG2 cells growing in galactose medium after KR treatment. HepG2 cells were treated with KR for 6 h, and alterations in Ψm were analyzed using the mitochondrial membrane potential (MMP)-sensitive cationic dye JC-1. Confocal microscopy (**a**) and flow cytometry (**b**) analyses revealed two populations of the cells with high (red) and low (green) Ψm, which correspond to aggregates and monomers of the dye, respectively. **c** The bar diagram shows the ratio of JC-1 monomers to aggregates under treatment condition. **d** A simultaneous analysis of mitochondrial redox state was performed by a TMRE fluorescence inner membrane potential probe. The mean fluorescence intensity was estimated and presented as bar plot ± SD. **e** Cellular ATP level was analyzed as a function of KR concentration in HepG2 cells growing in galactose medium. ATP depletion was estimated after 2 h of incubation with KR. Data are shown as mean ± SD from three independent experiments. Statistical significance is indicated with asterisks: (ns) p > 0.05, (*) p < 0.05, (**) p < 0.01, (***) p < 0.001, (****) p < 0.0001

The MMP indicates the vitality of the cell, and its reduction leads to apoptosis. We investigated the influence of KR on the changes in the mitochondrial redox state of HepG2 cells growing in galactose medium after 6 h treatment (Fig. 4). The cells were stained with JC-1, and the changes in Ψm were analyzed in parallel by flow cytometry and confocal microscopy (Fig. 4a–c). Untreated cells were used as a positive control, and CCCP was used to create a strong, single positive green fluorescence control. Cells with high MMP promote the formation of dye aggregates and emit red fluorescence. Cells with low MMP contain more JC-1 monomers and emit green fluorescence (Fig. 4a–c). Depolarization of the cell membrane is associated with the degradation of mitochondria and increased green fluorescence intensity. KR treatment led to a decrease in red fluorescence emitted by aggregates. Based on this finding, the ratio of monomers to aggregates (535/595) was evaluated (Fig. 4c). The higher the value of this ratio, the higher was the membrane depolarization observed. In HepG2 cells treated with 80 µM KR, the ratio increased 4.5-fold compared with that in control cells (Fig. 4b, c). Furthermore, simultaneous monitoring of TMRE (in the lower quenching mode) fluorescence confirmed that 6-h treatment with KR decreased the MMP, together with a decrease in 75% fluorescence intensity in 80 µM KR-treated cells (Fig. 4d). By using bioluminescent assay for the quantitative determination of ATP, we demonstrated depletion of cellular ATP level in HepG2 cells treated with KR (galactose medium). A rapid decrease in ATP level was detectable within 2 h, and dropped over 56% in the cells exposed to 80 µM KR, while the lowest concentration had no effect. Therefore the result shows the effect of the two highest concentrations. Optimal time conditions were chosen, and the extension of the incubation time did not result in higher depletion of ATP. Taken together, these data suggest that rapid and pronounced ATP depletion is an early consequence of KR exposure and leads to loss of cell viability (Fig. 4e).

### KR disrupts oxidative parameters in cells differentiated in galactose medium

**Fig. 5:**
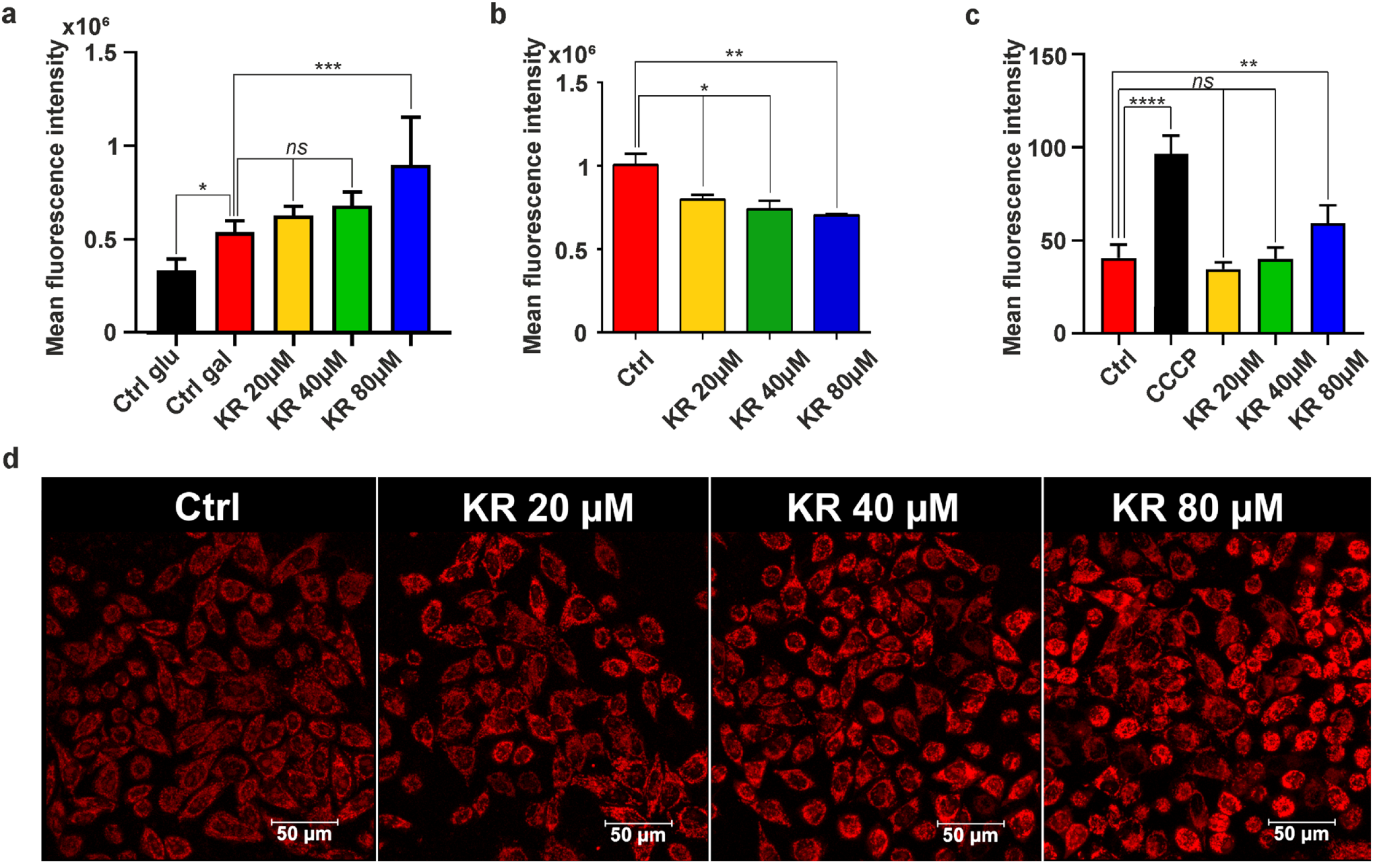
Galactose-forced OXPHOS enhances KR oxidative status deregulation. Reactive oxygen species (ROS) production was analyzed after KR treatment (6 h) of HepG2 cells cultured in galactose medium. **a** For intracellular ROS detection, the cells were stained with H_2_DCFDA, and the fluorescence intensity was measured by flow cytometry. Data are presented as a bar graph of three independent experiments (mean ± SD). **b** The level of cellular GSH was measured by staining with the nonfluorescent Thiolite™ Green dye that becomes strongly fluorescent upon reacting with thiols. Fluorescence intensity shift is plotted in a bar graph of three independent experiments (mean ± SD). **c** Flow cytometry and **d** confocal microscopy analyses of mitochondrial oxidative stress induction in HepG2 cells after KR treatment. Fluorescence intensity shift is presented as a representative histogram of three independent experiments and plotted in a bar graph (mean ± SD). Statistical significance is indicated with asterisks: (ns) p > 0.05, (*) p < 0.05, (**) p < 0.01, (***) p < 0.001

In the next step, we analyzed ROS production to confirm whether the pathological level disrupts the physiological state of cells by damaging proteins, lipids, and nucleic acids. Cytoplasmic ROS was measured by H_2_DCFDA staining using flow cytometry (Fig. 5a). The analysis of HepG2 cells growing in galactose medium and treated with increasing concentrations of KR for 6 h showed an increase in fluorescence emitted by the oxidized dye up to 37% at the highest concentration of KR, thus reflecting induction of oxidative stress. These results strongly suggest that oxidative stress induced by KR can trigger cell apoptosis (Fig. 5a). We also observed that KR treatment entailed reduction in the total GSH content, which is a natural component of the antioxidant defense system. The decrease (30% compared to control cells) in cellular GSH concentration was measured by staining with the nonfluorescent Thiolite Green dye (Fig. 5b).

We analyzed the mitochondrial oxidative stress induction by KR in HepG2 cells using flow cytometry and confocal microscopy (MitoSOX staining). Red fluorescence of the dye is triggered selectively in the mitochondria in the presence of superoxide. Our analysis revealed that 80 µM KR induces superoxide production in the cells (an increase of approximately 32% in fluorescence intensity was observed as compared to untreated cells, which reflected the increase in mitochondrial oxidative stress), whereas the effect was not apparent with lower concentration (Fig. 5c, d).

## Discussion

Recent studies on anticancer therapies based on low-molecular-weight compounds indicated that targeting defined molecular structures or the entire metabolic pathways strongly disrupts the functioning of cancer cells [21]. Such compounds have a specific mechanism of action, which is often very complex and may rely on the activation or inactivation of biological pathways associated with tumor regression. Low-molecular-weight compounds can also affect the activation of cellular machinery through direct effect on the mitochondria [22]. Application of small compounds, including derivatives of nucleosides, might be a promising therapeutic approach. It involves the activation of a cellular response to the stress and maintenance of metabolic and energetic balance within the cell [23, 24].

Mitochondria play a crucial role in the regulation of cellular energy metabolism, as they control ATP generation, redox homeostasis, and the apoptotic pathway. Mitochondria are an important element in the process of oncogenesis, through the flexible switch between glycolysis and OXPHOS. The switch in their metabolic phenotype ensures the survival of cancer cells and compensates for the high energy demand [25]. Recently, many strategies have been developed to target mitochondria, including compounds that disrupt electron transport chain, the apoptotic pathway, or ROS homeostasis [22]. Currently, the challenge is that cancer cells can also acquire a hybrid phenotype, in which both glycolysis and OXPHOS can be utilized. This phenotype facilitates metabolic plasticity of cancer cells more specifically in metastasis and therapy resistance [25]. Thus, discovering new therapies against mitochondrial impairment is very challenging, with many limitations associated with the susceptibility of cancer cells to drugs [26].

When considering the metabolism of cancer cells on a broader level, and not only from the mitochondrial level, it is essential to understand that this field is based on the principle that metabolic activities are altered in cancer cells relative to normal cells. These alterations support the maintenance of malignant properties of cancer cells [27, 28]. Currently, in the context of the altered metabolic activity of cancer cells, the following two concepts are considered: “metabolic reprogramming” and “oncometabolites.” The second one is a relatively new term and refers to the involvement of the metabolites in malignant phenotype. However, in this work, we focused on “metabolic reprogramming,” which includes metabolic pathways that are enhanced or suppressed by tumorigenic mutations and other factors such as an external agent [28]. The classical example of a reprogrammed metabolic pathway in cancer is the Warburg effect or aerobic glycolysis. It promotes increased anabolism, which includes the synthesis of nucleotides, amino acids, and lipids; alters antimetabolic stress responses to maintain hemostasis and survival; and affects gene expression in a metabolism-dependent manner to support their proliferation [29].

Cancer cells possess elevated glucose uptake ability and mainly depend on glycolysis, even in the presence of oxygen and functional mitochondria [13]. This phenomenon known as the Crabtree effect is one of the multiple metabolic strategies, but it ensures survival under adverse metabolic conditions [6]. Although glycolysis is relatively inefficient in generating ATP when compared with OXPHOS [5], it provides sufficient intermediates for the biosynthesis of nucleotides, NADPH, NADH, and amino acids [30]. One of the hypotheses suggests that the Crabtree effect occurs when glycolytic enzymes (phosphoglycerate kinase and pyruvate kinase) compete for free cytoplasmic ADP with the mitochondria [8]. It was shown that glucose medium (both high and low concentrations) provides an optimal environment for cancer cell proliferation and tumor invasion, but impairs the cytotoxicity of therapeutic drugs and prevents estimation of their efficacy [6, 31]. We also encountered this impediment in our research while testing compounds with a potential impact on mitochondrial dysfunction. Therefore, to overcome the limitations of the Crabtree effect and to study drug-induced mitochondrial toxicity, remodeling of energetic metabolism of cancer cells must be conducted. The common strategy to increase cancer cell toxicity is the replacement of glucose with galactose in the culture medium as the only oxidizable substrate [32]. Because cells cultured in galactose rely mostly on OXPHOS, they become more sensitive to mitochondrial toxicants [33]. Yet, this research strategy was tested solely in the context of well-established mitotoxicants, including respiratory chain inhibitors or drugs that cause the so-called drug-induced liver injury (mainly exhibiting antidiabetic function) [34]. Currently, significant developments in advanced technologies have enabled various discoveries in cancer metabolism, including quantifying metabolites (e.g., NMR analysis) or measuring the activities of metabolic pathways (isotope tracing studies). Taken together, it is possible to integrate it with functional genomics for better understanding of metabolic vulnerabilities in cancer cells [28]. Our studies focused on how an external compound affects cell function. Although we have not used the above mentioned technologies, our research may serve as an introduction to more detailed analyses.

Here, we show how a specific compound, N^6^-furfuryladenosine (KR), which is a purine derivative, affects cancer cell metabolism and its bioenergetics by interfering with purine metabolism (data not shown). By using the appropriate research model, i.e., HepG2 cells cultured in galactose environment, we attempted to prove that KR induces apoptosis by activating this intrinsic pathway directly related to the mitochondria.

Purine nucleoside analogs or antimetabolites are an important class of drugs used in the treatment of cancer. Antimetabolites are a family of compounds that are a mainstay in cancer chemotherapy, and their chemical structures are similar to either folate or nucleotides, which are the building blocks of DNA [35].

KR has been recently reported to have a strong anticancer effect, and it significantly disturbs the energy balance within the cells, induces cellular genotoxic stress, and shows potent antiproliferative and apoptogenic activity against various human cancer cell lines [36]. KR disrupts the MMP, induces the release of cytochrome *c*, and activates caspase-3 [37]. It was shown that the antiapoptotic Bcl-2 protein was downregulated, while the proapoptotic Bax protein was upregulated upon exposure to KR. KR has also been reported as a potent inhibitor of CCND2 whose transactivation leads to the downregulation of cyclins D1 and D2 [36]. These effects seem to be selective and promote cell death in cancer cells [38]. KR and several purine derivatives that are widely used in cancer treatment have a specific and unique mode of action, which may involve the activation of a cellular response to stress and maintenance of the metabolic balance of the cells [24]. The main mechanism of action of purine analogs leads to cell death and exclusively involves phosphorylated metabolites whose activity is dependent on adenosine kinase (ADK) and requires intracellular accumulation of mono-, di-, and tri-phosphates [39]. It was shown that the use of an ADK inhibitor completely inhibited KR-induced apoptosis and cytotoxicity, which suggests that cancer cell selectivity may be achieved based on the known overexpression of ADK by cancer cells. This also suggests that KR bioactivation through metabolic conversion into the nucleotide form is essential for its cytotoxicity [36]. Except for use in chemotherapy, KR and its ProTides activate the Parkinson’s Disease Associated PTEN-Induced Putative Kinase 1 (PINK1) independent of mitochondrial depolarization. This highlights the potential of the modified nucleosides and their phosphate prodrugs as treatment agents for neurodegenerative diseases [40].

In previous studies, known mitotoxicants, e.g., rotenone, oligomycin, or FCCP, have shown a stronger effect on cells growing in galactose medium, and a significant decrease in cell viability was observed as compared to that for cells growing in glucose medium [12]. Similar effects were observed with KR. We compared our research findings to those obtained in tumor cells treated with metformin, which is considered as a potential anticancer agent and chemotherapeutic adjuvant [30, 41]. Compared to KR, metformin has a different chemical structure, but studies in glucose-rich conditions also encountered several limitations. Glucose deprivation enhanced anticancer activity of metformin, leading to disruption of cell bioenergetics by decreasing ATP production and inhibiting survival signaling pathways [13, 30].

Here, we extended previous observations of the anticancer activity of KR. We performed a series of experiments comparing the effect of KR in HepG2 cells cultured in both glucose and galactose medium and showed its enhanced effect in conditions forcing OXPHOS. The Crabtree effect is not restricted only to cells cultured in high-glucose medium, but it also occurs in cells cultured in low-glucose medium [6]. This was also confirmed in our research. The basic culture medium for HepG2 cells is EMEM, which contains 1,000 mg/l of D-glucose and is already considered as a low-glucose medium. Thus, all the experiments designed by us to compare the behavior of cancer cells in the presence of glucose were conducted in this particular culture medium without differentiating between low- and high-glucose medium vs. galactose.

We confirmed that KR induces cell toxicity, inhibits cell proliferation, and initiates cell apoptosis in both glucose-deprived and glucose-enriched conditions, with greater and faster effect in the galactose environment (Fig. 1a, b). This is also confirmed by reduction in the number of mitochondria in KR-treated cells co-cultured in galactose, because mitochondrial bioenergetic capacity is closely correlated with mitochondrial morphology and pathology (Fig. 1c, d) [42]. To confirm that HepG2 cells cultured in galactose medium are forced to utilize OXPHOS, we performed cell energy phenotype analysis using a Seahorse Analyzer. We proved that galactose metabolism is unable to ensure sufficient amount of ATP from glycolysis; thus, the increase in oxygen capacity was observed in cells that predominantly rely on OXPHOS. We compared the values of OCR and ECAR in both culture environments, which indicated a higher potential of cells for ECAR in glucose and higher utilization of oxygen in galactose (Fig. 1f, g).

By using a glucose analog, 2-NBDG, we showed that its appearance in galactose medium immediately forced the cells to undergo glycolysis. Glucose uptake by cells reflected increasing fluorescence, which was proportional to increasing KR concentrations [43]. This indicates that 2-NBDG uptake can be used to measure changes in glycolysis, and its potential can be used in early phases of drug development (Fig. 1e) [44]. This suggests that the cells actively undergo the Crabtree effect when cytotoxic factors appear in the cellular environment.

The above results demonstrate that the viability of HepG2 cells after KR treatment is strictly related to the induction of cell death via the intrinsic apoptotic pathway (Fig. 2e); thus, all subsequent experiments were performed exclusively using the galactose-enriched medium. First, to compare the effect of KR in cells, we analyzed apoptosis/necrosis induction in different cell lines that exhibited the Crabtree effect and proved that glucose withdrawal enhances the toxicity of KR. It was also shown that the observed effect depends on cell type (Fig. 2a–d). A previous study reported that increased cell death of breast cancer cells occurred when they were treated with metformin in the absence of glucose [45].

Drugs that affect cell energy metabolism are considered to be promising agents in anticancer treatment [46]. Among these drugs, 2-DG, an inhibitor of hexokinase (HK), is a rate-limiting enzyme of glycolysis via inhibition of its first reaction, and it interferes with anabolic reactions [2, 47]. It was reported that 2-DG might decrease oxygen consumption and abolish the Crabtree effect [48, 49]. On the other hand, based on Warburg’s observations, 2-DG cannot be considered as a monotherapeutic agent because of its temporary influence as most cancer cells rely on OXPHOS [2]. Treatment with 2-DG reduces the antioxidant potential of cancer cells and induces an energetic stress via the reduction of TCA flux [50].

We observed that the strategy based on sugar replacement with subsequent KR treatment is more effective in inducing apoptosis, rather than inhibition of glycolysis with 2-DG and simultaneously treating cells with KR (Fig. 3a, b). The use of 2-DG might be a confirmation that KR affects the mitochondrial cell death pathway; however, the mechanism of action of 2-DG in cancer cells is very complex and needs further investigation. Direct inhibition of glycolysis was less efficient than the enhancement of OXPHOS rate in the cells, but still, agents that target both glycolysis and OXPHOS function hold promise as an ideal anticancer therapeutic approach. A recent study showed that combining metformin with 2-DG depletes ATP and exhibits synergistic therapeutic effect on pancreatic cancer cells [22].

The analysis of changes in the MMP was performed by flow cytometry and confocal microscopy (Fig. 4a–d). Similar to the results obtained from the apoptosis/necrosis assay, we investigated the influence of KR on changes in the mitochondrial redox state in HepG2 cells growing in galactose medium. We observed a strong effect of KR action, which caused depolarization of the mitochondrial membrane (Fig. 4a–d). This may be because these cells have a large number of mitochondria and are therefore best suited for the analysis of processes affected by low-molecular-weight compounds [34].

It is known that KR can disrupt the energy balance in cells through phosphorylation by ADK, which is crucial for KR toxicity [8, 21, 51, 52]. We also showed that in response to KR treatment in galactose medium, HepG2 cells undergo extensive apoptosis, which is likely a direct consequence of the rapid decrease in the cellular ATP level (Fig. 4e). It was found that low glucose level and metformin treatment of cancer cells lead to cell death by decreasing ATP production [47], and these results were also observed during cell treatment with 2-DG co-cultured in glucose withdrawal conditions [15]. This indicates that KR reduced the cellular ATP level, which might be correlated with disturbances in respiratory state associated with ATP synthesis and needs further investigation. A similar response was observed with inhibitors of the electron transport chain [12]. Currently, there are other ADK-dependent ATP-depleting adenosine derivatives such as 2-chloro- and 8-chloroadenosine [53], which interfere with ATP synthesis. They act through structure-based inhibition of mitochondrial ATP synthase.

Finally, we proved that KR in galactose medium triggers cell death via apoptosis, not only by disruption of cellular ATP generation (Fig. 4e), but also by induction of oxidative stress (Fig. 5a, b). ROS are intracellular chemical species that contain oxygen and include superoxide anion (O_2_^−^), hydrogen peroxide (H_2_O_2_), and hydroxyl radical (OH^·^). Cancer cells have a high level of oxidative stress [28] that is exerted by the accumulated ROS, which is induced by hypoxia, metabolic defects, and endoplasmic reticulum stress [41]. Cancer cells can maintain the expression of ROS scavengers (antioxidant proteins) at a constant high level in response to elevated concentrations of free radicals. Thus, promoting mitochondrial ROS production to induce cancer cell death may enhance the activity of chemotherapy [20]. In response to KR-induced energetic stress, the byproducts of oxygen metabolism, intracellular ROS, are formed (the level of intracellular ROS generation was analyzed by dual staining with H_2_DCFDA/PI), with significant depletion of reduced glutathione in HepG2 cells (Fig. 5b). We observed that glucose deprivation and its replacement with galactose (Fig. 5a) elevates ROS level itself by diminishing their degradation along with increasing their generation. Glucose in culture medium is essential for ROS detoxification [2], but increasing KR concentration disrupts this process and further enhances its cytotoxicity (Fig. 5a). Therefore, the mitochondrial production of ROS was also analyzed (Fig. 5c, d). We observed the increase in superoxide level when KR concentration increased. ROS production by the mitochondria leads to mtDNA damage and mutations, which in turn might lead to progressive respiratory chain dysfunction and to further increase in ROS production as a consequence of this dysfunction [54].

We showed that the alterations in glucose metabolism and the appearance of an external agent, i.e., KR, disrupt the proper functioning of the cells and affect cytosolic and mitochondrial redox potential and ATP generation [55]. It seems that observations such as increasing oxidative stress and the simultaneous reduction in the MMP are contradictory. However, several compounds such as capsaicin, casticin, and myricetin show this mechanism of action and exhibit anticancer activity by increasing ROS generation, leading to the disruption of the mitochondrial transmembrane potential in cancer cells [22]. Many ETC inhibitors such as metformin, tamoxifen, α-tocopheryl succinate (α-TOS), and 3-bromopyruvate (3BP) act via disrupting the induction of high ROS level.

By using complementary cell-based assays, we investigated the effect of KR on mitochondrial functions. The increased toxicity in cancer cells by glucose withdrawal may also show that the multiple pathways affected by KR enhance one another to promote cancer cell death. Our studies demonstrate that this metabolic model serves as an effective tool to elucidate mitochondrial dysfunctions after compound treatment of HepG2 cells. The utilization of galactose, which forces OXPHOS and bypasses the Crabtree effect, is a methodological approach that allows better screening of drugs targeting the mitochondria.

Our findings suggest that metabolic plasticity of most cancer cells is a challenge in testing putative drugs that induce mitochondrial impairment. KR might elicit disorders in energy deficit of cancer cells, although glucose withdrawal itself is obviously not a viable therapeutic approach. Creating an in vivo model in this manner is impossible, because dietary galactose will not affect the circulation glucose level and cannot be manipulated as that in cell culture. However, there have been proposals for potential clinical approaches that mimic similar conditions through the simultaneous use of the inhibitors of the PI3K/Akt pathway and metformin [56].

Our experimental pipeline was not designed to test the clinical effect of KR but to show that galactose in culture medium makes cells more reliant on OXPHOS (oxygen consumption doubles in galactose medium) and thereby become susceptible to potential mitochondrial toxicants. In addition, we used HepG2 cells with appropriate characteristics for in vitro experiments, and these cells are better suited for the assessment of drug-induced mitochondrial dysfunction.

Although there are a few FDA-approved cancer-directed therapies that specifically target mitochondria and evasion of apoptosis, these therapies have shown promising results in clinical trials in a variety of cancers [57, 58]. The present study helped to understand the mechanism of cancer cell resistance for KR treatment. Our results indicate that KR affects many cellular parameters (inhibition of proliferation, oxidative stress, cell energy phenotype, and induction of apoptosis), resulting indirectly in mitochondrial stress and disruption of energetic metabolism. We proved that purine derivatives can also be an effective mitotoxicant and might be potentially used to exploit the metabolic vulnerability of cancer cells.

We have certainly shown an indirect effect of KR on the mitochondria, and such a fast path provides opportunities to further explore this topic by analyzing the cascade of enzymatic reactions that are associated inter alia with the activation of oncogenic pathways. This comprehensive analysis showed that all mechanisms involved in mitochondrial impairment, such as disruption of oxidative parameters, mitochondrial membrane depolarization, reduced ATP level, and induction of apoptosis, are the result of the action of KR. On the basis of our present research, we confirmed that purine derivatives might be potentially used to exploit the metabolic vulnerability of cancer cells.

## Data Availability

Data can be provided upon request.

## Abbreviations

2-DG: 2-Deoxy-D-glucose
2-NBDG: 2-(N-(7-nitrobenz-2-oxa-1,3-diazol-4-yl) amino)-2-deoxyglucose
7-AAD: 7-Aminoactinomycin D
A172: Human glioblastoma cell line
ADK: Adenosine kinase
ADP: Adenosine diphosphate
AICAR: 5-Aminoimidazole-4-carboxamide 1-β-d-ribofuranoside
ATP: Adenosine triphosphate
BAX: Bcl-2-associated X protein
BCl-2: B cell lymphoma 2
CCCP: Carbonyl cyanide 3-chlorophenylhydrazone
CCND2: Cyclin D2
DAPI: 2-(4-Amidinophenyl)-1H -indole-6-carboxamidine
DMEM: Dulbecco’s Modified Eagle Medium
DPBS: Dulbecco’s phosphate-buffered saline
ECAR: Extracellular acidification rate
EMEM: Eagle’s Minimum Essential Medium
FBS: Fetal bovine serum
FCCP: Carbonyl cyanide-4-(trifluoromethoxy)phenylhydrazone
FDA: Food and Drug Administration
GSH (2S): 2-Amino-4-{[(1R)-1-[(carboxymethyl)carbamoyl]-2-sulfanylethyl]carbamoyl}butanoic acid; glutathione
H_2_DCFDA: 2ʹ,7ʹ-Dichlorodihydrofluorescein diacetate
HEPES: 4-(2-Hydroxyethyl)-1-piperazineethanesulfonic
HepG2: Human liver hepatocellular carcinoma cell line
JC-1: 5,5ʹ,6,6ʹ-Tetrachloro-1,1ʹ,3,3ʹ-tetraethylbenzimidazolylcarbocyanine iodide
KR: Kinetin riboside
MMP: Mitochondrial membrane potential
MtDNA: Mitochondrial DNA
NADH: Reduced form of micotinamide adenine dinucleotide
NADPH: Nicotinamide adenine dinucleotide phosphate
OCR: Oxygen consumption rate
OXPHOS: Oxidative phosphorylation
PBS: Phosphate-buffered saline
PI: Propidium iodide
ROS: Reactive oxygen species
T47D: Human mammary gland cell line
TMRE: Tetramethylrhodamine
